# Respiratory Infection with Enterovirus Genotype C117, China and Mongolia

**DOI:** 10.3201/eid2006.131596

**Published:** 2014-06

**Authors:** Zichun Xiang, Sosorbaramyn Tsatsral, Chunyan Liu, Linlin Li, Lili Ren, Yan Xiao, Zhengde Xie, Hongli Zhou, Guy Vernet, Pagbajabyn Nymadawa, Kunling Shen, Jianwei Wang

**Affiliations:** MOH Key Laboratory of Systems Biology of Pathogens, Beijing, China; (Z. Xiang, L. Li, L. Ren, J. Wang);; Institute of Pathogen Biology, Beijing (Z. Xiang, L. Li, L. Ren, Y. Xiao, H. Zhou, J. Wang);; National Center of Communicable Diseases, Ulaanbaatar, Mongolia (S. Tsatsral, P. Nymadawa);; Beijing Children’s Hospital Affiliated to Capital Medical University, Beijing (C. Liu, Z. Xie, K. Shen);; Fondation Mérieux, 69365 Lyon, France (G. Vernet);; Mongolian Academy of Medical Sciences, Ulaanbaatar (P. Nymadawa)

**Keywords:** EV-C117, Picornaviridae, picornavirus, enterovirus, rhinovirus, viruses, respiratory tract infection, children, pediatric, China, Mongolia

**To the Editor:** Enteroviruses (EVs) are small, nonenveloped viruses of the family *Picornaviridae* (*1*). EVs are classified into 12 species according to the molecular and antigenic properties of their viral capsid protein (VP1). To date, 7 species are known to infect humans, including EV-A to EV-D and rhinovirus A, B, and C (http://www.picornastudygroup.com/taxa/species/species.htm)

EV-C117 was a newly found EV-C genotype. It was identified in a nasopharyngeal sample from a hospitalized child, 3 years and 9 months of age, with community-acquired pneumonia in Lithuania in 2012 (*2*,*3*). However, aside from this case, little is known about the prevalence and clinical significance of EV-C117. Here, we report the detection of EV-C117 in children in China and Mongolia with respiratory tract infections (RTIs).

During March 2007–March 2013, we screened for EV-C117 in respiratory samples from patients with RTIs in China and Mongolia, including nasopharyngeal aspirates collected from 3,108 children in China who had lower respiratory tract infections when they were admitted to Beijing Children’s Hospital (*4*) and swab samples from 2,516 patients in Mongolia with influenza-like illness (Technical Appendix Table 1). Respiratory viruses in samples from China were screened by using multiplex PCR and single PCR assays as described (*4*). Samples from Mongolia were screened by using the FTD Respiratory Pathogens Multiplex Assay Kit (Fast-track Diagnostics, Luxembourg City, Luxembourg). EV-positive samples were further genotyped by using reverse transcription PCR (RT-PCR) and primers sequentially targeting the VP1 region (*5*), the 5′-untranslated region (5′-UTR)/VP4/VP2 region (*6*) and the 5′-UTR (*7*). A 394-nt amplicon corresponding to the 5′-UTR of EVs was obtained from 10 children in China; a 598-nt amplicon corresponding to the 5′-UTR/VP4/VP2 region was obtained by RT-PCR from 5 children in Mongolia. Blastn analysis (http://www.blast.ncbi.nlm.nih.gov/Blast.cgi) of PCR amplicons showed that only amplicons detected in 2 children from China (patients BCH096A and BCH104A) and 2 children from Mongolia (patients MGL126 and MGL208) had the highest similarity (95%–98%) to the EV-C117 prototype strain LIT22.

To further confirm that these 4 strains belong to EV-C117, we attempted to amplify the full-length viral genome sequences. However, we only obtained full-length viral genome sequences from the 2 strains found in patients from China (GenBank accession nos. JX560527 [patient BCH096A], and JX560528 [patient BCH104A], respectively). For the remaining 2 strains from Mongolia, MGL126 (5′UTR/VP4/VP2: KF726102; VP1: KF726100) and MGL208 (5′UTR/VP4/VP2: KF726103; VP1: KF726101), we obtained the sequence of the 5′-UTR/VP4/VP2 region and VP1 gene. Phylogenetic analysis of these sequences showed that they all belonged to genotype EV-C117 (Figure, panels A and B).

**Figure:**
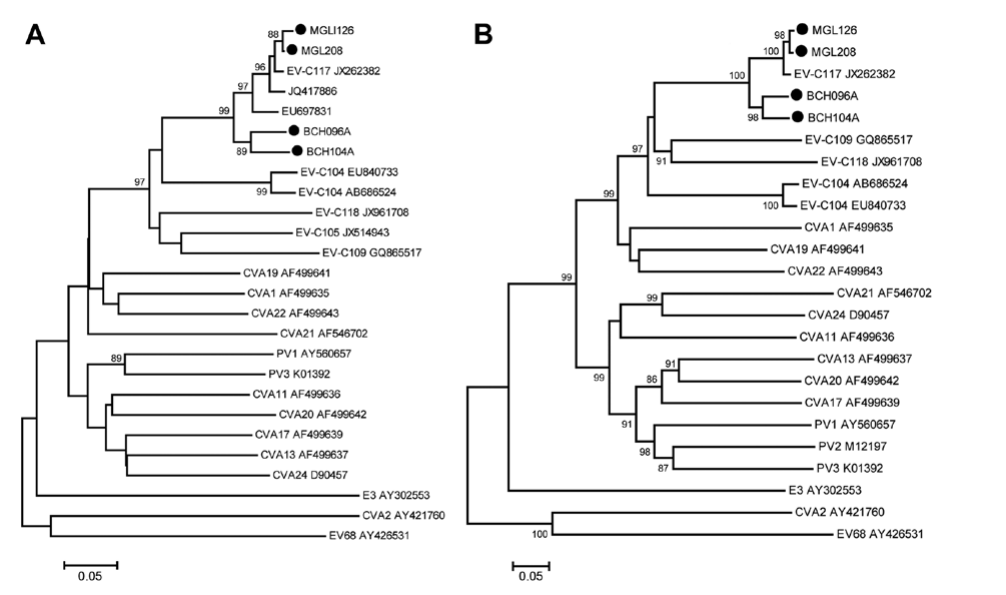
Phylogenetic analysis of enterovirus genotype C117 (EV-C117) based on nucleotide sequences. Phylogenetic trees were generated with 1,000 bootstrap replicates. Neighbor-joining analysis of the targeted nucleotide sequence was performed by using the Kimura 2-parameter model with Molecular Evolutionary Genetics Analysis (MEGA) software version 4.0 (http://www.megasoftware.net). The EV-C117 strains identified in this study are indicated by black circles. Enterovirus 68, cocksackievirus (CV) A2, and echovirus (E) 3 (GenBank accession nos. AY426531, AY421760, and AY302553) were used as outgroups. PV, poliovirus. A) Phylogenetic analysis of the VP 4/VP2 region (399 nt, corresponding to nt 673–1,071 of EV-C117 prototype strain LIT22 [JX262382]). B) Phylogenetic analysis of the viral protein1 region (888 nt, corresponding to nt 2416–3303, numbered according to the sequence of LIT22). Scale bars represent nucleotide substitutions per site.

Virus isolation for EV-C117 by using Vero and H1-HeLa cells was unsuccessful. Through blastn and phylogenetic analyses, we also found that the previously identified EV-C strain HC90835 (EU697831, from Nepal) (*8*), EV-C104 strain CL-C22 (EU840734, EU840744, and EU840749, from Switzerland) (*9*) and a rhinovirus strain SE-10–028 (JQ417886, from South Korea), also belong to EV-C117 (Figure, panel A), indicating that EV-C117 is widely distributed geographically. Because a large proportion of EV infections are subclinical or mild (*1*), the prevalence of EV-C117 should be further estimated by using serologic investigations in general populations.

The VP1 sequences of the EV-C117 strains isolated in China and Mongolia were 89.9%–95.6% (nt) and 95.2%–98.3% (aa) identical to the EV-C117 prototype strain LIT22 (patient JX262382). Alignment analysis of amino acid sequences showed differences between strains isolated in this study and LIT22, i.e., Ser^15^ (strains in this study) versus Asn^15^ (LIT22). In addition, we found that the strains from patients in China contain Lys^63^ and Ala^90^, and those from Mongolia have Thr^93^, Asn^97^, and Ser^276^. The biological significance of these mutations is unknown.

Of these 4 EV-C117–positive children, 3 were hospitalized with respiratory disease (Technical Appendix Table 2); the nonhospitalized child (MGL208) had a sore throat, but no other signs or symptoms. The viral loads of EV-C117 and co-detected viruses were quantified by using real-time PCR (methods available upon request), with a median EV-C117 load of 2.9 × 10^5^ RNA copies/mL (range 1.1–4.8 × 10^5^ RNA copies/mL [Technical Appendix Table 2]). EV-C117 was the only virus detected in patients BCH104A and MGL126. Respiratory syncytial virus (5.0 × 10^6^ copies/mL) and rhinovirus (1.5 × 10^5^ copies/mL) were detected in patient BCH096A, and influenza virus A (IFVA, H3N2; 5.1 × 10^10^ copies/mL) and human bocavirus (3.7 × 10^2^ copies/mL) were detected in patient MGL208.

The co-detection of viruses in 2 of the EV-C117–positive patients raises the question of what role EV-C117 plays in RTIs. However, it is notable that EV-C117 was the only virus detected in the other 2 patients. This finding indicates that, at least in patients with low resistance (patient BCH104A had severe bacterial infection before EV-C117 was detected and patient MGL126 had congenital heart disease), EV-C117 might be associated with RTIs. In addition, the strain isolated in Nepal and the strain isolated in Switzerland, EV-C117, were both detected in specimens collected from patients with RTIs (*8*,*9*). Collectively, these data indicate the respiratory tropism of EV-C117. Additional epidemiologic and virologic studies on EV-C117 may be warranted to establish its role in RTIs.

Technical Appendix Molecular diagnosis and characteristics of patients screened for EV-C117.

## References

[1] Pallansch M, Roos R. Enteroviruses: polioviruses, coxsackieviruses, echoviruses, and newer enteroviruses. In: Knipe DM, Howley PM, editors. Field’s virology. 5th ed. Philadelphia: Lippincott Williams & Wilkins; 2007.

[2] Daleno C, Piralla A, Scala A, Baldanti F, Usonis V, Principi N, Complete genome sequence of a novel human enterovirus C (HEV-C117) identified in a child with community-acquired pneumonia. J Virol. 2012;86:10888–9 . 10.1128/JVI.01721-12 22966184 PMC3457295

[3] Daleno C, Piralla A, Usonis V, Scala A, Ivaskevicius R, Baldanti F, Novel human enterovirus C infection in child with community-acquired pneumonia. Emerg Infect Dis. 2012;18:1913–5 . 10.3201/eid1811.120321 23092590 PMC3559168

[4] Xiang Z, Xie Z, Wang Z, Ren L, Xiao Y, Li L, Human enterovirus genotype 104 infection in China. Emerg Infect Dis. 2013;19:689–91. 10.3201/eid1904.121435 23750592 PMC3647420

[5] Nix WA, Oberste MS, Pallansch MA. Sensitive, seminested PCR amplification of VP1 sequences for direct identification of all enterovirus serotypes from original clinical specimens. J Clin Microbiol. 2006;44:2698–704. 10.1128/JCM.00542-06 16891480 PMC1594621

[6] Savolainen C, Blomqvist S, Mulders MN, Hovi T. Genetic clustering of all 102 human rhinovirus prototype strains: serotype 87 is close to human enterovirus 70. J Gen Virol. 2002;83:333–40 . 11807226 10.1099/0022-1317-83-2-333

[7] Lee WM, Kiesner C, Pappas T, Lee I, Grindle K, Jartti T, A diverse group of previously unrecognized human rhinoviruses are common causes of respiratory illnesses in infants. PLoS ONE. 2007;2:e966. 10.1371/journal.pone.0000966 17912345 PMC1989136

[8] Briese T, Renwick N, Venter M, Jarman RG, Ghosh D, Köndgen S, Global distribution of novel rhinovirus genotype. Emerg Infect Dis. 2008;14:944–7. 10.3201/eid1406.080271 18507910 PMC2600308

[9] Tapparel C, Junier T, Gerlach D, Van-Belle S, Turin L, Cordey S, New respiratory enterovirus and recombinant rhinoviruses among circulating picornaviruses. Emerg Infect Dis. 2009;15:719–26. 10.3201/eid1505.081286 19402957 PMC2687021

