# Supplementary material for: Respiratory Infection with Enterovirus Genotype C117, China and Mongolia
Source: Emerg Infect Dis. 2014 Jun;20(6):1075–7. doi: 10.3201/eid2006.131596 (PMC4036781; doi:10.3201/eid2006.131596)
Supplement: Technical Appendix — Molecular diagnosis and characteristics of patients screened for EV-C117. [file 13-1596-Techapp-s1.pdf]

Article DOI: http://dx.doi.org/10.3201/eid2006.131596

Respiratory Infection with Enterovirus Genotype C117, China and Mongolia

Technical Appendix tables

Technical Appendix Table 1. Molecular diagnosis and characteristics of patients screened for EV-C117, China and Mongolia*

Characteristics	China	Mongolia
Patients, no.	3,108†	2,516
Type of sample	Nasopharyngeal aspirates	Swab
Period of sample collection	Mar. 2007–Feb. 2012	Nov. 2008–Mar. 2013
Male	1,963	1, 244
Female	1,145	1,272
Age range	9 d–14 y	0–86 y
Median age	1 y	2 y
Total no. of EV positive	37 (24 male, 13 female)†	55 (35 male, 20 female)
Total no. of EV-C117 positive	2	2

*EV-C117, enterovirus C117.

†This data has been reported in a previous study (*4*).

Technical Appendix Table 2. Characteristics of 4 patients in whom EV-C117 was detected

Patient identification no.	BCH096A	BCH104A	MGL126	MGL208
Age (mo)/sex	3/F	23/M	27/F	36/F
Date of admission	Jul. 2007	Jul. 2007	Nov. 2011	Jan. 2013
Diagnosis or clinical symptoms	Asthmatic bronchitis, rhinorrhea, sneezing, expectoration	Pneumonia, lung abscess, purulent pleurisy, fever (39.3°C), cough	Lobar pneumonia, cough, fever (39.5°C), expectoration	Sore throat
Comorbid conditions	None	Bacterial infection	Congenital ventricular septal defect	None
Virus and viral load (RNA or DNA copies/mL)	EV-C117: 1.1×10^5<br>RSVA: 5.0×10^6<br>RV: 1.5×10^5	EV-C117:4.4×10^5	EV-C117:1.3×10^5	EV-C117:4.8×10^5<br>IFVA:5.1×10^{10}<br>HBoV:3.7×10^2
GenBank no.	Full length: JX560527	Full length: JX560528	VP1: KF726100<br>5 [illegible]<br>KF726102	VP1: KF726101<br>5 [illegible]<br>KF726103

*EV-C117, enterovirus C117; RSV, respiratory syncytial virus; RV, human rhinovirus; IFVA, human influenzavirus A; HBoV, human bocavirus.
